# Dynamic Evolution of EEG Complexity in Schizophrenia Across Cognitive Tasks

**DOI:** 10.3390/e27030226

**Published:** 2025-02-22

**Authors:** Rosa Molina, Yasmina Crespo-Cobo, Francisco J. Esteban, Ana Victoria Arias, Javier Rodríguez-Árbol, Maria Felipa Soriano, Antonio J. Ibáñez-Molina, Sergio Iglesias-Parro

**Affiliations:** 1 Department of Psychology, University of Jaén, 23071 Jaén, Spain; rmlopez@ujaen.es (R.M.); aibanez@ujaen.es (A.J.I.-M.); siglesia@ujaen.es (S.I.-P.); 2 Department of Experimental Biology, University of Jaén, 23071 Jaén, Spain; festeban@ujaen.es; 3 Department of Social Psychology, National Distance Education University (UNED), 28040 Madrid, Spain; avarias@psi.uned.es; 4 Mental Health Department, San Agustín University Hospital, 33401 Linares, Spain; mariaf.soriano.sspa@juntadeandalucia.es

**Keywords:** schizophrenia, electroencephalography, Higuchi fractal dimension, neural complexity, temporal dynamics

## Abstract

Schizophrenia is characterized by widespread disruptions in neural connectivity and dynamic modulation. Traditional EEG analyses often rely on static or averaged measures, which may overlook the temporal evolution of neural complexity across cognitive demands. This study employed Higuchi Fractal Dimension, a non-linear measure of signal complexity, to examine the temporal dynamics of EEG activity across five cortical regions (central, frontal, occipital, parietal, and temporal lobes) during an attentional and a memory-based task in individuals diagnosed with schizophrenia and healthy controls. A permutation-based topographic analysis of variance revealed significant differences in neural complexity between tasks and groups. In the control group, results showed a consistent pattern of higher neural complexity during the attentional task across the different brain regions (except during a few moments in the temporal and occipital regions). This pattern of differentiation in complexity between the attentional and memory tasks reflects healthy individuals’ ability to dynamically modulate neural activity based on task-specific requirements. In contrast, the group of patients with schizophrenia exhibited inconsistent patterns of differences in complexity between tasks over time across all neural regions. That is, differences in complexity between tasks varies across time intervals, being sometimes higher in the attentional task and other times higher in the memory task (especially in the central, frontal, and temporal regions). This inconsistent pattern in patients can explain reduced task-specific modulation of EEG complexity in schizophrenia, and suggests a disruption in the modulation of neural activity on function of task demands. These findings underscore the importance of analyzing the temporal dynamics of EEG complexity to capture task-specific neural modulation.

## 1. Introduction

The brain’s complex dynamics are inherently non-linear, with non-linearity arising even at the cellular level due to threshold and saturation phenomena in neuronal behavior [1]. These non-linear properties extend to large-scale neural networks, where interactions among neurons give rise to emergent behaviors that cannot be fully captured by linear models. Electroencephalogram (EEG) signals are particularly valuable in neuroscience research due to their high temporal resolution, which enables the capture of rapid neural dynamics on the millisecond scale. This precision is crucial for studying the brain’s non-linear and transient processes, as cognitive functions such as attention, memory, and sensory integration often unfold over very short time intervals [2]. Historically, EEG analyses have heavily relied on linear methods focused on spectral power or averaged measures across time and frequency domains. While these approaches have provided critical insights into brain function, they often fail to capture the full complexity and temporal variability of neural processes, particularly those underlying cognitive tasks [3,4].

To address these limitations, non-linear methods have emerged as powerful tools for quantifying the irregularity and complexity of EEG signals. These measures are particularly adept at capturing dynamic changes in brain activity that occur during both physiological states and pathological conditions. For instance, fractal dimension analysis has been applied to study the spatiotemporal complexity of brain activity in Alzheimer’s disease [5], epilepsy [6], and schizophrenia [4]. Such methods provide unique insights into how neural networks adapt—or fail to adapt—to cognitive demands. Higuchi’s fractal dimension is a nonlinear measure used to quantify the complexity of time-series data, such as EEG signals. This method evaluates the self-similarity and irregularity of a signal over multiple scales, providing a robust representation of its dynamic properties. Unlike linear methods, Higuchi’s fractal dimension is particularly well-suited for nonstationary data, as it captures the intricate temporal patterns inherent in brain activity without requiring transformations into the frequency domain [7,8].

In the context of schizophrenia, EEG studies have consistently highlighted widespread disruptions in neural connectivity and dynamic modulation, particularly during tasks requiring attention or memory retrieval [9,10]. These disruptions are thought to stem from impairments in the brain’s ability to dynamically reconfigure its functional networks in response to changing cognitive demands. However, many traditional EEG studies rely on static or averaged measures, which may obscure subtle but meaningful temporal fluctuations in neural complexity that are critical for understanding these impairments. For example, averaged power spectral analyses or event-related potentials (ERPs), while informative, often fail to capture the non-linear and transient nature of brain activity [2,11].

Recent advances in non-linear time-series analysis have provided powerful tools for exploring the temporal dynamics of brain activity. Unlike linear methods, which assume proportionality and constant relationships between variables, non-linear methods capture irregularities and emergent behaviors characteristic of brain activity [1]. HFD quantifies the fractal complexity of EEG signals directly in the time domain, offering a sensitive measure of how neural complexity evolves dynamically over time [7]. This method is particularly well-suited for studying schizophrenia, where abnormalities in neural complexity have been linked to core cognitive deficits, such as impaired working memory and attentional control [4,12]. A study of Iglesias-Parro et al. [13] showed that schizophrenic patients, when compared with control participants, exhibited less differentiated EEG complexity at different conscious states (external attention vs. mind wandering). This finding suggests that patients tend to show the same overall complexity pattern regardless of the specific mental state that they present. In the present study, we aim to explore whether this reduction of differentiation holds for attention and memory processes and whether a temporal evolution of complexity analysis adds new light on understanding EEG complexity patterns in schizophrenic patients.

The attention task consisted of presenting images with local landscapes to participants, and they needed to detect whether a small red dot appeared on the screen. In the memory task, the same type of images were presented, but in this case, participants were instructed to recall an event from their memory. Hence, our study builds on this growing body of research by applying HFD to analyze the temporal evolution of EEG complexity across different cortical regions during attentional (Dots) and memory-based (Remember) tasks in individuals diagnosed with schizophrenia (SCZ) and healthy controls (CTRL). By focusing on temporal evolution rather than averaged measures, we aim to capture the dynamic modulation of neural networks underlying task-specific processing. We hypothesize that individuals with schizophrenia will exhibit reduced task-specific modulation of EEG complexity and less consistent patterns of neural complexity over time compared to controls. This approach highlights the importance of moving beyond traditional static analyses to better understand the disrupted dynamics of brain activity in schizophrenia.

In this study, we conceptualize complexity as the brain’s capacity to generate diverse, adaptive, and organized patterns of activity. This concept aligns with theoretical frameworks that describe the brain as a dynamic system operating at the edge of order and chaos. Such a balance is essential for functional efficiency and cognitive flexibility, allowing neural networks to adapt dynamically to changing cognitive demands. By employing Higuchi’s fractal dimension, we aim to quantify this balance and explore how it varies across tasks (Dots vs. Remember) and groups (controls vs. schizophrenia). The primary objective of this study was to identify significant differences in brain activity between the experimental conditions Dots and Remember using Higuchi’s complexity metric. These differences were analyzed across temporal windows (Steps) and brain regions (topologies) using a permutation-based topographic analysis of variance (TANOVA). This approach was inspired in [14], allowing for robust statistical testing of differences while accounting for the spatial and temporal structures of EEG data. To complement the TANOVA findings, the correlation between the complexity of different brain regions across tasks (Dots and Remember) was analyzed in both the control and schizophrenia groups.

## 2. Method

### 2.1. Participants

The sample included 21 individuals diagnosed with schizophrenia (SCZ) attending the Mental Health Day Unit at the University St. Agustin Hospital (Spain) and 17 healthy controls (HC).

In the SCZ group, 19% were women and 81% were men. The mean age was 36.476 (*SD* = 8.256, *median*: 34 [*min*: 25, *max:* 51]). For the SCZ participants, the inclusion criteria were an ICD-10 diagnosis of schizophrenia (F20) or a psychotic disorder (F23). The educational level of the participants was categorized into three categories: basic (primary and secondary education), medium (general certificate of education, certificate of higher education), and high (university degree). According to these categories, the sample of participants with SCZ included 57.1% participants with a basic educational level, 28.6% with a medium level, and 14.3% with a high level.

In the HC group, 41% of participants were women and 59% were men. The mean age was 41.7 (*SD* = 13.1, *median*: 38 [*min*: 25, *max*: 66]). This group included 35.3% with a basic educational level, 41.2% with a medium level, and 23.5% with a high level.

There was neither a significant association between group and education level (*χ*^2^ (4, *N* = 38) = 6.209, *p* = 0.184), nor between sex and group (*χ*^2^ (1, *N* = 38) = 2.237, *p* = 0.135), nor did the groups differ significantly with respect to age (*t* = 1.500; *p* = 0.142).

Exclusion criteria for all groups were as follows: concurrent diagnosis of neurological disorder, concurrent diagnosis of substance abuse, history of developmental disability, inability to sign informed consent, or vision disorders (vision disorders which, although corrected by glasses or contact lenses, suppose a loss of visual acuity, e.g., cataracts). In addition, an exclusion criterion for the HC group was the diagnosis of a mental disorder (according to verbal reports from participants). All participants gave their written informed consent according to the Declaration of Helsinki and the Ethics Committee on Human Research of the Hospital approved the study.

### 2.2. Procedure

The experiment was carried out in one session in the laboratory of the Mental Health Department at San Agustín Hospital in Linares. All participants provided written informed consent before participating in the study, in accordance with the principles outlined in the Declaration of Helsinki. The study protocol was approved by the Human Research Ethics Committee of the hospital. Participants were seated at a distance of approximately 70 cm from a computer screen. A set of 64 active electrodes was then placed on the participants’ scalps, with electrode impedance maintained below 5 kΩ.

Participants in this study performed two tasks while their EEG activity was recorded with an AD rate of 500 Hz. The experimental tasks were carried out in the same order for all participants. First, they performed a sustained attention task, and subsequently, within the same experimental session and after a 5 min break, they completed a recall task.

In the sustained attention task (Dots) participants were shown a series of neutral images (landscapes) on a computer screen. Each image was displayed for 10 s. In 50% of the trials, a red dot appeared randomly at any location on the screen. Participants were instructed to press a designated key as soon as they detected the red dot. The task comprised 40 trials in total: 20 trials in which the red dot was present and 20 trials without it. The trials with a dot and without a dot were presented in random order. Following the dot detection task, participants completed a memory task (Remember). During this phase, the same set of images was presented again on the screen for 10 s each. Participants were instructed to observe each image and allow their thoughts to wander freely to any memory, regardless of whether it was related to the content of the image. This task consisted of 20 trials.

Each trial was divided into 40 temporal windows of 2 s each, referred to henceforth as steps. Each step overlapped 90% of the time with the previous one. These steps represent consecutive time intervals during which Higuchi’s fractal dimension (HFD) was calculated for EEG signals recorded from the 63 electrode channels referenced to the Cz electrode site. The trials were grouped by experimental conditions (Dots and Remember), and the electrodes were further categorized into five brain regions or topologies: frontal, central, occipital, parietal, and temporal. Below is a detailed description of how the channels were assigned to each topology (see Appendix A).

The frontal topology included 16 electrodes positioned over the anterior region of the scalp. These regions have been associated with higher-order cognitive functions such as decision-making, attention, and working memory [15]. The central topology comprised 13 electrodes located near the central midline of the scalp. This region is primarily involved in motor control and somatosensory processing [16]. This grouping ensured that activity from both hemispheres and the central sulcus was adequately represented. The parietal topology included 16 electrodes positioned over the parietal cortex, which is associated with sensory integration, spatial reasoning, and attention [17]. The occipital topology consisted of 8 electrodes placed over the posterior region of the scalp, corresponding to areas responsible for visual processing [18]. The temporal topology included 10 electrodes located over the lateral temporal regions of the scalp. These regions are involved in auditory processing, language comprehension, and memory functions [19,20].

The assignment of the channels to topologies was based on standard EEG electrode placement systems [10,11,12,13,14,15,16,17,18,19,20] and their correspondence to underlying cortical regions. This grouping facilitated region-specific analyses while maintaining consistency with established neuroanatomical landmarks [21].

### 2.3. Data Processing, Analysis, and Results

Data processing was conducted using Brain Vision Analyzer (Version 2.2.2., Brain Products GmbH, Gilching, Germany), EEGLAB (Version 2023.0, Swartz Center for Computational Neuroscience, San Diego, CA, USA), and MATLAB (R2024b, MathWorks, Natick, Massachusetts, MA, USA). Blinks and other artifacts were removed using Infomax ICA (version 2.2.0.0, https://www.mathworks.com/matlabcentral/fileexchange/38300-pca-and-ica-package (accessed on 5 May 2018)). The Infomax ICA algorithm is based on the principle of maximizing entropy. Specifically, it seeks to optimize the representation of the data by finding an unmixing matrix that maximizes the mutual independence of the sources. Unlike other dimensionality reduction techniques, such as principal component analysis (PCA), which decorrelates data but assumes orthogonality, Infomax ICA focuses on achieving statistical independence without imposing constraints on orthogonality. ICA components containing artifacts were identified by visual inspection of the scalp topography, power spectra, and raw activity from all components. Once the noisy components were selected, they were removed from the original signals.

The cleaned EEG signals were then used as inputs for a custom MATLAB script developed to calculate Higuchi’s fractal dimension (HFD) as a measure of signal complexity over time. This non-linear approach provides insight into brain activity dynamics by capturing variations in signal complexity [7]. We selected HFD due to its computational efficiency and relevance for analyzing nonstationary signals. This metric was chosen over other nonlinear measures, such as entropy-based methods or recurrence quantification analysis (RQA) because it provides a direct and interpretable measure of signal complexity while being computationally less demanding. HFD is a measure of irregularity for discrete time series. The algorithm obtains new series by sampling the original signal at different intervals (k). For each k, the lengths, L(k), of the signals are calculated, normalizing the sums of the differences of the values, with a distance of k and a starting point m (m = 1, 2,…, k). Finally, a double logarithmic plot, ln L(k) vs. ln k, is used to estimate the actual dimension value of the signal. The range of values for HFD lies between 1 and 2. Higher HFD values indicate greater EEG complexity, often associated with increased neural activity, greater neural network engagement, and higher cognitive demands. Conversely, lower HFD values reflect more regular or predictable activity, typically observed during resting states or under reduced cognitive load. For this experiment, we calculated a single HFD estimation for each trial using a sliding window procedure (*n* = 40 windows or steps).

To statistically evaluate the differences in neural complexity between experimental tasks and groups, we implemented linear mixed-effects models (LMMs) using the R package lme4 (version, 1.1-36 R Core Team, Global). Separate models were fitted for healthy controls and individuals with schizophrenia to account for group-specific neural dynamics. Each model incorporated Task (Dots vs. Remember) and Topology (brain region: central, frontal, occipital, parietal, temporal) as fixed effects, along with their interaction term (Task × Topology), to assess how task-related differences in complexity varied across cortical regions. To account for repeated measures and individual variability, random intercepts were included for participants (ID), allowing baseline complexity levels to vary between individuals. Additionally, random slopes for Task nested within ID were incorporated to model intra-individual variability in task-related complexity differences.

Next, to examine how task-related neural complexity evolved across both cortical regions and temporal windows, we implemented a permutation-based topographic analysis of variance (TANOVA) inspired by [14]. This method extends traditional ANOVA by explicitly modeling the interaction between Task (Dots vs. Remember) and Topology (brain regions) across successive temporal steps (1–40), thereby capturing spatiotemporal dynamics often obscured in static analyses based on averages across time.

HFD values were calculated for 40 steps separately for each of the five brain regions. To assess statistical significance, condition labels (Dots/Remember) were permuted 1000 times within each Step–Topology pair, generating a null distribution of differences. Observed differences were considered significant if they exceeded the 95th percentile of this distribution (*p* < 0.05, two-tailed). The TANOVA’s design allowed us to test whether task effects depended on specific spatiotemporal contexts (i.e., Task × Topology × Step interactions), a critical feature for detecting dynamic reconfigurations in neural networks.

To control for Type I error inflation from multiple comparisons (40 steps × 5 topologies = 200 tests per group), we applied Benjamini–Hochberg false discovery rate (FDR) correction (*q* < 0.05) [22]. This approach ensured the robust identification of regions and time points where task demands significantly modulated neural complexity while preserving the hierarchical structure of EEG data across space and time.

To further explore the relationships between brain regions and to complement the findings from the TANOVA, we conducted correlation analyses. These analyses were performed to assess the strength and direction of functional connectivity between regions (central, frontal, occipital, parietal, and temporal) during the Dots and Remember tasks for both the control and schizophrenia groups. By calculating pairwise correlations of complexity values across regions, we aimed to capture patterns of synchronization and interaction that could provide additional insights into task-specific network dynamics and group differences.

We have structured our analyses and results into two main sections. First, we present dynamic analyses that explicitly consider the temporal dimension of complexity differences between experimental tasks for controls. Second, we introduce the equivalent analyses for the participants with schizophrenia.

#### 2.3.1. Control Participants

A linear mixed-effects model (LMM) was conducted to evaluate differences in EEG complexity (Higuchi fractal dimension) between the attentional and memory-based tasks in the healthy control participants. The model included fixed effects for Task, Topology, and their interaction, with random intercepts and slopes for Task nested within participants to account for individual variability.

The analysis revealed a non-significant main effect of Task, *F*(1, 16) = 2.27, *p* = 0.151, indicating no overall differences in neural complexity between the tasks across participants. However, a significant main effect of Topology was observed, *F*(4, 2,608,158) = 1858.04, *p* < 0.001, showing pronounced variability in complexity across brain regions. Importantly, the interaction between Task and Topology was statistically significant, *F*(4, 2,608,158) = 44.86, *p* < 0.001, indicating that task-related differences in complexity were region-specific.

**Figure 1:**
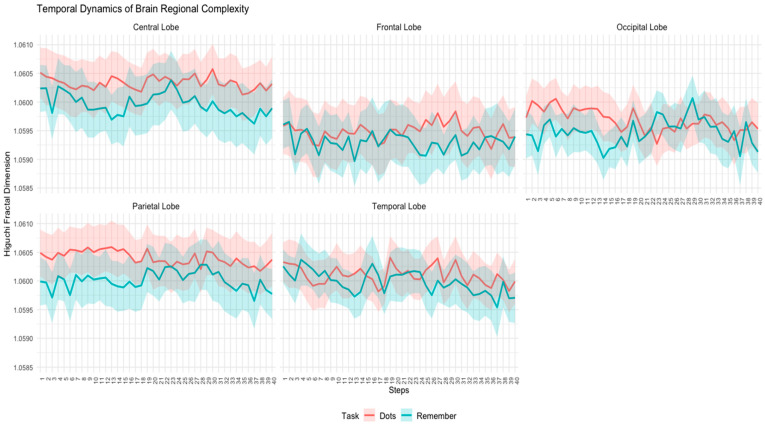
Temporal dynamics of Higuchi’s fractal dimension across brain regions (central, frontal, occipital, parietal, and temporal lobes) for the control group during attentional (Dots) and memory (Remember) tasks. The solid lines represent the mean HFD values, while the shaded areas indicate the standard error of the mean (SEM).

For each topology, we computed the mean Higuchi fractal dimension across all subjects and trials for each task (Dots and Remember). These mean values illustrate the temporal evolution of complexity within a typical trial. The results are shown in Figure 1.

Next, HFD values were calculated for 40 steps separately for each of the five brain regions. These differences provided a measure of the observed effect size for each temporal window and brain region.

A permutation-based approach was employed to test the statistical significance of the observed differences. Condition labels (Dots and Remember) were randomly shuffled within each experimental condition (Step and Topology). For each permutation, the mean difference was recalculated. This process was repeated 1000 times to generate a null distribution of differences. The null distribution allowed us to estimate the likelihood that the observed differences occurred by chance.

HFD values were calculated for 40 steps separately for each of the five brain regions, and *p*-values were calculated as the proportion of permuted differences that were greater than or equal to the absolute value of the observed difference. To control for Type I error due to multiple comparisons, the false discovery rate (FDR) correction was applied using the Benjamini–Hochberg procedure [22].

**Figure 2:**
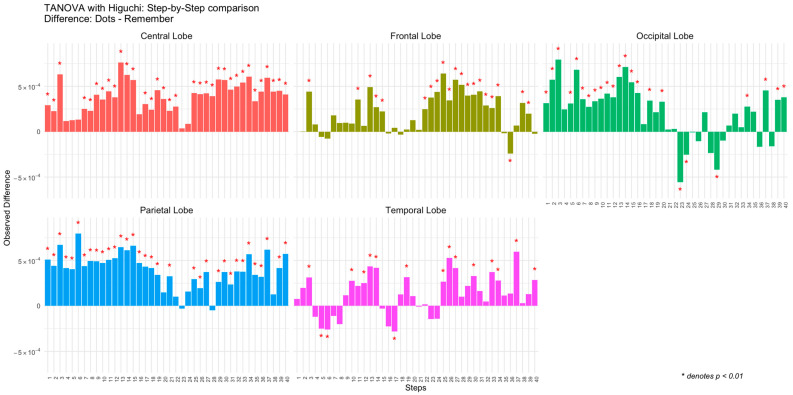
Differences in observed Higuchi complexity between tasks (Dots vs. Remember) for the control group across brain regions (central, frontal, occipital, parietal, and temporal lobes) and steps.

The results can be visualized as bar plots, with each bar representing the observed difference for a specific combination of Step and Topology (see Figure 2). Significant differences were highlighted with red asterisks above or below the corresponding bars, depending on the sign. The visualization included separate panels for each brain region (Topology).

As can be seen in Figure 1 and Figure 2, the control participants exhibited a consistent temporal pattern of higher complexity during the attentional task compared to the memory task. This is especially evident in the central, frontal, and parietal regions. In the occipital and temporal regions, there were a few specific moments when complexity was higher during the memory task, but for most periods, complexity was higher in the attentional task.

#### 2.3.2. Participants with Schizophrenia

Replicating the mixed model framework used for the controls, patients exhibited a non-significant main effect of Task, *F*(1, 20) = 0.007, *p* = 0.931, indicating no overall differences in neural complexity between the attentional and memory tasks across participants. However, a significant main effect of Topology was observed, *F*(4, 3,283,510) = 3648.86, *p* < 0.001, showing substantial variability in complexity across brain regions. Importantly, the interaction between Task and Topology was statistically significant, *F*(4, 3,283,510) = 57.598, *p* < 0.001, highlighting that task-related differences in complexity depended on specific brain regions.

**Figure 3:**
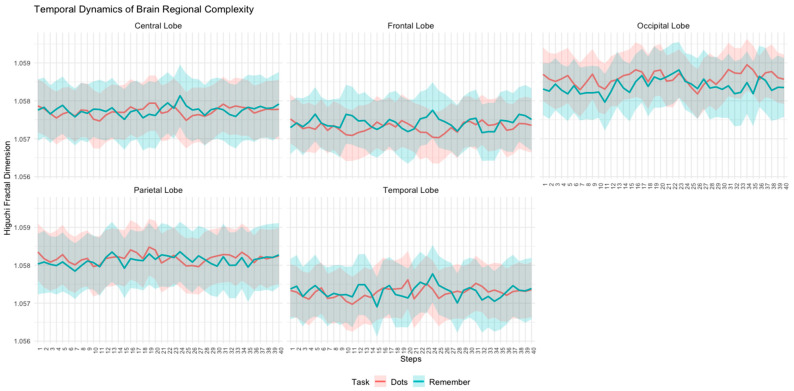
Temporal dynamics of Higuchi’s fractal dimension across brain regions (central, frontal, occipital, parietal, and temporal lobes) for the schizophrenia group during attentional (Dots) and memory (Remember) tasks. The solid lines represent the mean HFD values, while the shaded areas indicate the standard error of the mean (SEM).

Next, we replicated the same permutation-based topographic analysis of variance (TANOVA) methodology previously applied to the control group to examine the temporal evolution of Higuchi’s fractal dimension (HFD) during task performance across brain regions. Specifically, for each topology, the mean Higuchi fractal dimension across all subjects and trials was computed for each task (Dots and Remember). The results for this sample are presented in Figure 3.

Next, HFD values were calculated for 40 steps separately for each of the five brain regions.

**Figure 4:**
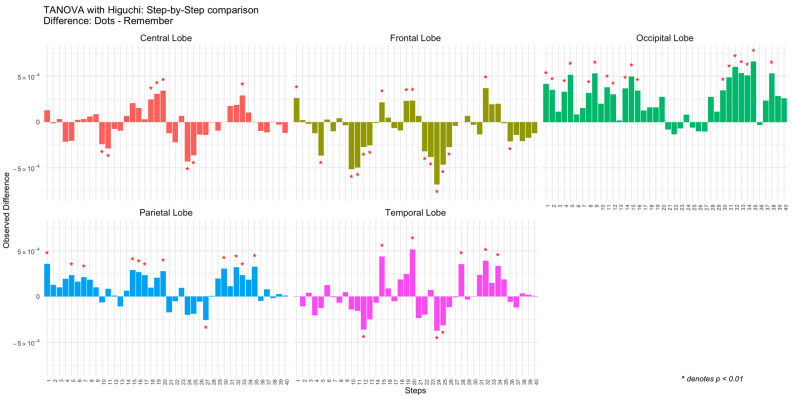
Differences in observed Higuchi complexity between tasks (Dots vs. Remember) for the schizophrenia group across brain regions (central, frontal, occipital, parietal, and temporal lobes) and steps.

The results are presented as bar plots (Figure 4), where each bar represents the observed difference for a specific combination of Step and Topology. Significant differences are marked with red asterisks. As with the control group, separate panels are included for each brain region (Topology) to facilitate comparison. Positive differences indicate greater complexity during the attentional task (Dots), while negative differences reflect greater complexity during the memory task (Remember).

**Figure 5:**
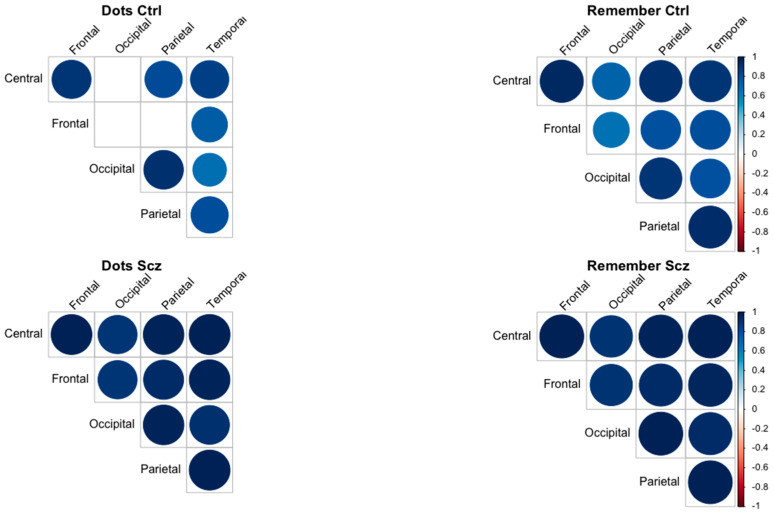
Correlation matrices between brain regions (central, frontal, occipital, parietal, and temporal) based on complexity values during the Dots and Remember tasks in control (Ctrl) and schizophrenia (Scz) groups.

In Figure 3 and Figure 4, the patterns of complexity differences between tasks in the patient group demonstrate considerable variability. Specifically, in all brain regions, the complexity values during the attentional task (Dots) are higher than those observed during the memory task (Remember) at certain temporal steps, while at other steps, the opposite occurs (Figure 5). This temporal variability does not follow a consistent or statistically significant pattern across time. The inconsistency is particularly pronounced in the central, frontal, and temporal regions, where fluctuations between tasks appear more irregular and lack a clear trend. These findings suggest that, in patients, the dynamic modulation of brain complexity between tasks is less structured compared to what might be expected in a more organized neural system.

## 3. Discussion

In this study, we explored the temporal dynamics of EEG complexity during an attentional and memory task in healthy controls and patients with schizophrenia. Previous research has shown that patients with schizophrenia exhibit reduced task-specific modulation of EEG complexity compared to healthy controls [13]. These findings suggest that patients exhibit a diminished ability of the brain to dynamically adjust its functioning to meet the cognitive demands of the task. In this experimental design, we aimed to further explore brain dynamics by analyzing the temporal evolution of complexity across different cognitive tasks.

Consistent with our hypothesis, patients and controls showed differences in the temporal patterns of EEG complexity. Healthy controls exhibited a highly regular temporal pattern, showing increased complexity during the attentional task consistently over time. However, in the group of patients, differences in complexity between tasks varied inconsistently over time. This variability likely accounts for the reduced complexity differences between tasks reported in previous research; unpredictable fluctuations in patients’ EEG complexity levels (sometimes higher, sometimes lower) result in a smaller average difference between tasks.

To our knowledge, this finding of high temporal inconsistency in complexity among patients is entirely novel. It supports the notion that patients with schizophrenia have impaired modulation of brain complexity in response to task demands and struggle to adjust brain dynamics to the task at hand.

Now, we will discuss these differences between patients and controls according to the brain regions of interest (central, frontal, occipital, parietal, and temporal lobes).

### 3.1. Central, Parietal, and Frontal Lobe

We have grouped the discussion of the results into these regions since the findings are similar across them both in the controls and patients. The CTRL group consistently shows significant positive differences favoring the attentional task. This result may be reflective of efficient somatosensory integration and motor control, as well as executive high-order processes, necessary for attentional tasks [23]. In contrast, the SCZ group exhibits inconsistent patterns of neural complexity differences between tasks, with both positive and negative differences observed. The presence of negative differences in the SCZ group, which are absent in the CTRL group, suggests that individuals with schizophrenia may struggle to maintain stable neural complexity across tasks. This variability could reflect compensatory mechanisms or disruptions in connectivity that impair their ability to dynamically adapt to cognitive demands [9,12]. The presence of negative differences in the SCZ group is particularly intriguing because it indicates greater neural complexity during the memory compared to the attentional task, an atypical pattern not observed in the CTRL group. This may suggest that individuals with schizophrenia require additional neural engagement during memory-based tasks to compensate for deficits in episodic memory retrieval or sensory integration. Such findings align with prior research indicating that schizophrenia is associated with altered connectivity and reduced efficiency in neural networks responsible for attention and memory [9,16]. As we have mentioned before, these findings support the idea that with schizophrenia there is a disruption in neural modulation, leading to both reduced task-specific modulation of EEG complexity and unexpected shifts in cortical complexity. Further investigation into these negative differences could provide valuable insights into the compensatory mechanisms at play in schizophrenia and how they influence cognitive performance.

### 3.2. Occipital and Temporal Lobes

In the occipital and temporal lobes, the temporal pattern of differences was more similar in the controls and patients. In the control group, negative and positive differences in complexity could be observed, especially in temporal regions. That is, in some temporal steps there was higher complexity in the attentional task, and in others, there was higher complexity in the memory task, possibly reflecting the role of temporal regions in episodic memory retrieval through interactions with the hippocampus and prefrontal cortex [24,25]. Patients with schizophrenia also showed negative and positive differences although again, the pattern was more irregular and variable.

On the whole, our findings support prior research indicating that schizophrenia is associated with deficits in sensory integration and attentional control [9,21,26]. It is reasonable to think that this inability to modulate brain activity on the function of the task contributes to difficulties prioritizing external stimuli versus internal representations, which are key deficits observed in schizophrenia [26].

## 4. Limitations

The study has certain limitations that should be considered when interpreting the results. The relatively small sample size may limit the generalizability of the results to broader populations. Future studies with larger and more diverse samples could provide additional insights and strengthen the conclusions drawn here. Additionally, the experimental tasks were performed in a fixed order for all participants, which might introduce potential order effects, such as fatigue or learning (although the temporal pattern during each task did not show fatigue or learning effects). While Higuchi’s fractal dimension (HFD) is a robust tool for analyzing EEG complexity, it is worth noting that it provides an overall measure of signal complexity without distinguishing between specific neural processes. The division of trials into 40 temporal windows allowed for detailed temporal analysis but may not fully capture rapid fluctuations in neural complexity at finer scales. Exploring alternative methods with even higher temporal resolution could complement these findings in future research. Finally, EEG inherently has limited spatial resolution compared to imaging techniques like fMRI. Although this study focused on cortical regions using well-established electrode groupings, future investigations combining EEG with other neuroimaging modalities could provide a more comprehensive understanding of neural dynamics. Despite these considerations, the study’s innovative use of HFD to explore temporal dynamics in schizophrenia offers valuable insights into neural complexity and task-specific modulation. In addition to HFD, future studies could benefit from incorporating other nonlinear and dynamic measures to analyze EEG complexity in schizophrenia. For instance, indices such as approximate entropy, multiscale entropy, or nonlinear correlation coefficients may provide complementary insights into the underlying neural dynamics.

## 5. Conclusions

Our results highlight significant differences in the dynamics of cortical activity between the CTRL and SCZ groups across various brain regions, emphasizing how schizophrenia disrupts task-specific neural modulation. In the CTRL group, cortical regions, such as the central, frontal, occipital, parietal, and temporal lobes, demonstrate dynamic and efficient neural responses tailored to the demands of attention-focused and memory-based tasks. These findings align with prior research indicating that healthy individuals exhibit robust neural flexibility, allowing them to allocate resources appropriately for sensory integration, attentional control, and episodic memory retrieval [23,24,25].

In contrast, the SCZ group shows reduced task-specific modulation of EEG complexity across all cortical regions analyzed. This diminished task-specific modulation reflects impairments in sensory integration, attentional control, and memory retrieval—core cognitive deficits associated with schizophrenia. For example, the parietal lobe shows fewer significant differences between tasks in patients with schizophrenia, suggesting difficulties in integrating multisensory information and directing attention effectively [10,27]. The frontal lobe also exhibits reduced modulation in SCZ participants, likely due to disrupted connectivity with other cortical regions such as the hippocampus, key for episodic memory retrieval and contextual integration [24,26].

The correlation analyses also support the hypothesis of a lack of functional specificity in the schizophrenia group. Thus, in the control group, distinct correlation patterns emerge depending on the task, reflecting functional specificity. During the Dots task, stronger correlations are observed between frontal and parietal regions, while the Remember task shows higher synchronization involving occipital and temporal regions. In contrast, the schizophrenia group exhibits consistently high correlations across all regions and tasks, suggesting a generalized hyperconnectivity pattern. This lack of task-dependent variability would indicate impaired functional segregation in the schizophrenia group.

Overall, these findings underscore widespread disruptions in neural networks in schizophrenia, highlighting the critical role of connectivity impairments in cognitive dysfunctions observed in this population.

The findings of this study could have significant clinical implications. Temporal differences in cortical activity may serve as neurophysiological biomarkers for the diagnosis of schizophrenia, the assessment of disease progression, and the evaluation of treatment response [28,29]. Identifying reliable biomarkers is essential for improving early detection and personalized treatment approaches. Therefore, future research should focus on integrating multimodal biomarkers to enhance diagnostic accuracy and optimize therapeutic strategies for schizophrenia. Likewise, these findings could serve as a starting point for future studies on other mental disorders, expanding the understanding of neurophysiological mechanisms beyond schizophrenia.

## Data Availability

The dataset supporting this study has been deposited in FigShare and is publicly available for other researchers. The dataset can be accessed via the following DOI: 10.6084/m9.figshare.28304096 (https://figshare.com/ndownloader/files/52005371, accessed on 10 September 2020).

## Appendix A

**Electrodes assigned to each topology**

Frontal Topology

Fp1, Fp2, AF7, AF3, AFz, AF4, AF8, F7, F3, Fz, F4, F8, F1, F5, F6, F2.

Central Topology

FC5, FC1, FCz, FC2, FC6, C3, C4, C1, C5, C2, FC3, C6, FC4.

Parietal Topology

CP5, CP1, CPz, CP2, CP6, P3, P4, Pz, P1, P2, P5, P6, P7, P8, CP3, CP4.

Occipital Topology

O1, Oz, O2, PO7, PO3, POz, PO4, PO8.

Temporal Topology

T7, T8, TP9, TP10, FT9, FT10, FT7, FT8, TP7, TP8.
